# Application of a SPOC-based blended teaching system in full-cycle training for medical imaging interns: A randomized controlled educational intervention study

**DOI:** 10.1097/MD.0000000000049908

**Published:** 2026-07-24

**Authors:** Junjie Chen, Jun Cui, Xin Li, Jiuxiao Yang, Xiao Yang, Hui Zhong, Fei Wang, Qingwen Gu, Cailiang Duan

**Affiliations:** a Deyang People’s Hospital, Deyang, Sichuan, China; b Akesu Prefecture First People’s Hospital, Akesu, Xinjiang, China.

**Keywords:** full-cycle training, medical imaging interns, Small Private Online Course (SPOC)

## Abstract

This single-center, parallel-group randomized controlled educational intervention study evaluated the short-term outcomes of an integrated, multicomponent teaching package combining a Small Private Online Course (SPOC)-based blended teaching system with full-cycle training among medical imaging interns. Sixty eligible interns undertaking clinical placements in the Department of Interventional Radiology at Deyang People’s Hospital between May 2023 and May 2024 were randomly assigned in a 1:1 ratio to the experimental or control group using a random number table (30 per group). The control group received conventional clinical internship teaching, whereas the experimental group received a package comprising the SPOC platform, micro-video modules, online quizzes, face-to-face seminars, case-based learning, structured instructor feedback, and stage-specific full-cycle training. Outcomes included self-reported perceived job competency, self-directed learning ability, teaching satisfaction, and department-specific end-of-rotation examination performance. Prior academic performance and digital literacy were assessed at baseline. At the single immediate post-intervention assessment, the experimental group reported higher perceived job competency, self-directed learning ability, and teaching satisfaction and achieved higher examination scores than the control group. However, several self-reported outcomes approached the upper limits of their scales. The findings therefore indicate favorable short-term between-group differences in perceived educational outcomes and department-specific examination performance. Because all outcomes were assessed only once immediately after the intervention, the study does not establish sustained learning, long-term knowledge retention, or meaningful improvement in subsequent clinical performance. In addition, because multiple educational components were implemented concurrently, the findings reflect the overall effect of the integrated package and cannot be attributed to the SPOC platform or any single component. Faculty development outcomes were not assessed. Multicenter studies with longitudinal follow-up and institutions differing in faculty capacity, teaching resources, and digital infrastructure are needed to evaluate durability, generalizability, implementation feasibility, resource requirements, workload, cost, and scalability.

## 1. Introduction

Medical imaging is a discipline that integrates theoretical knowledge, clinical practice, and applied diagnostic skills. For imaging students, the internship period is a critical stage in the transition from classroom-based learning to clinical work and from medical student to physician.^[1]^ During this period, students are expected to consolidate professional knowledge, acquire basic imaging and procedural skills, interpret common imaging findings, and develop diagnostic reasoning that aligns with clinical decision-making.^[2]^ However, medical imaging interns often face challenges such as insufficient clinical diagnostic thinking, inadequate practical skills, weak foundations in image interpretation, difficulty managing complex cases, and limited learning motivation.^[3]^ Therefore, improving clinical teaching models is essential to enhance interns’ engagement, initiative, and competency development. The “14th Five-Year Plan for National Clinical Specialty Capacity Building,” issued by the National Health Commission in October 2021, emphasizes the need to strengthen key specialties, including medical imaging, and to improve specialty capacity across different regions.^[4]^ In this context, cultivating high-quality medical imaging professionals has become an important task for the development of imaging education in China.

At present, teaching hospitals provide various forms of internship training according to their institutional conditions and medical imaging training requirements. Nevertheless, several limitations remain, including incomplete implementation of internship plans, insufficient diversity in teaching content and format, and limited individualization of training.^[5]^ Traditional internship teaching is still largely teacher-centered, with interns often acting as passive recipients of knowledge rather than active participants in learning. In addition, conventional face-to-face teaching may conflict with clinical schedules, resulting in poor attendance, difficulty organizing centralized sessions, and increased occupation of personal rest time. These problems may reduce interns’ willingness to participate and make it difficult to ensure consistent teaching quality.^[6]^

With the development of internet-based education and modern educational technologies, blended teaching based on Small Private Online Courses (SPOCs) has gradually emerged as a new teaching approach.^[7]^ This model combines online learning activities, such as micro-videos and online quizzes, with offline activities, including flipped classrooms, seminars, and case-based discussions. Compared with traditional teaching models, SPOC-based blended teaching offers greater flexibility in learning time and location, supports personalized learning, and facilitates teacher–student interaction and process-based learning management.^[8]^ Previous educational practices have suggested that SPOCs may help improve student engagement and provide learners with a more individualized learning experience.^[9]^ In China, some teaching hospitals have begun to explore SPOC-based models in medical education, including course preparation, online–offline instructional design, and course evaluation.^[10]^ However, its application in clinical internship training for medical imaging remains limited.

The characteristics of SPOC-based teaching, including small-scale implementation, restricted access, flexible learning, and individualized instruction, are compatible with the needs of medical imaging internship training. In this study, we designed an integrated SPOC-based blended teaching system combined with full-cycle training. The intervention incorporated synchronous online sessions, micro-video learning, online quizzes, offline seminars, case-based discussion, and practical training, covering medical imaging knowledge, basic interventional skills, communication skills, and scientific thinking. In addition to this, the training content was organized according to different stages of the internship, including preparation, core instruction, and consolidation, to provide stage-specific and targeted teaching support. This single-center randomized controlled educational intervention study was therefore conducted to evaluate the overall short-term educational outcomes of an integrated, multicomponent teaching package combining SPOC-based blended learning with stage-specific full-cycle training. The package incorporated digital learning resources, online assessment, face-to-face seminars, case-based learning, practical training, and structured instructor feedback. The study was not designed to determine the independent effect of the SPOC platform or any individual intervention component.

## 2. Methods

### 2.1. Study design and participants

**Figure 1.**
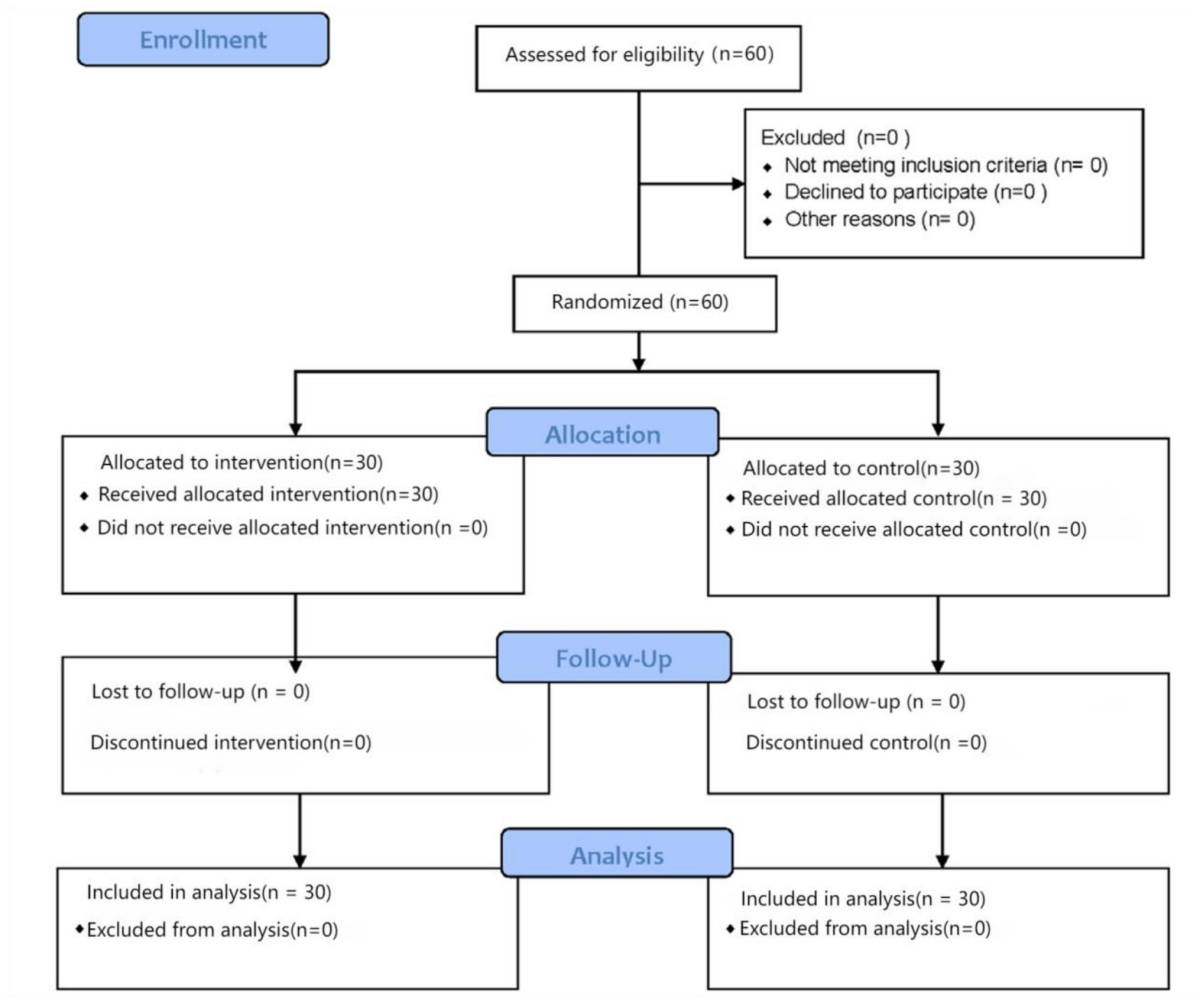
Study flowchart.

This was a single-center, parallel-group randomized controlled educational intervention study conducted in the Department of Interventional Radiology at Deyang People’s Hospital between May 2023 and May 2024. A total of 60 eligible medical imaging interns were enrolled and randomly assigned to either the experimental group or the control group in a 1:1 ratio, with 30 participants in each group. Randomization was performed using a random number table by an independent researcher who was not involved in teaching implementation or outcome assessment. To ensure allocation concealment, group assignments were placed in sealed opaque envelopes, which were opened only after participant enrollment. Because of the nature of the educational intervention, participants and instructors could not be blinded to group allocation. The 2 senior physicians who scored the practical skills component of the end-of-rotation examination were blinded to group allocation. The self-report outcomes were completed directly by participants and were therefore not assessor-blinded. The participant flow diagram is shown in Figure 1.

To ensure baseline equivalence between the 2 groups, we assessed 2 key indicators: prior academic performance (average score of professional courses during undergraduate studies) and digital literacy (using a simplified digital literacy scale for medical students, which includes 10 items covering basic computer operation, online resource search, and online learning platform use, with a total score of 10–50 points). The results showed no statistically significant differences between the 2 groups in prior academic performance (experimental group: 82.5 ± 3.2; control group: 81.9 ± 3.5; t = 0.325, *P* = .746) and digital literacy (experimental group: 42.3 ± 2.8; control group: 41.8 ± 3.1; t = 0.512, *P* = .610). No statistically significant between-group differences were observed in prior academic performance or digital literacy at baseline.

All methods were carried out in accordance with relevant guidelines and regulations. The experimental protocols have been approved by the Ethics Committee of the People’s Hospital of Deyang City (No. 2023-03-065-K01). Written informed consent was obtained from all participants in accordance with the Declaration of Helsinki.

### 2.2. Sample size

No formal a priori sample size or power calculation was performed. The sample size was determined by the number of eligible medical imaging interns available during the predefined recruitment period from May 2023 to May 2024. A total of 60 eligible interns were enrolled and randomly assigned in a 1:1 ratio. Accordingly, this study should be regarded as a small, exploratory randomized educational intervention study, and the precision and generalizability of the effect estimates should be interpreted cautiously.

### 2.3. Inclusion and exclusion criteria

#### 2.3.1. Inclusion criteria

Participants must be interns specializing in medical imaging; interns must have a clinical internship period of more than 3 months; interns must voluntarily agree to participate in this study and sign the informed consent form.

#### 2.3.2. Exclusion criteria

Resident physicians and visiting physicians; mid-study withdrawal; incomplete questionnaires (>5%).

### 2.4. Research methods

The control group followed the traditional clinical internship teaching model. Supervising instructors conducted teaching activities according to the clinical teaching plan and curriculum guidelines for the medical imaging program. The teaching content covered 2 main areas: fundamental knowledge of imaging and interventional radiology, and interventional procedural skills. Teaching was delivered using a “work-and-teach” approach, integrating clinical tasks with direct supervision and instruction from teaching staff.

**Figure 2.**
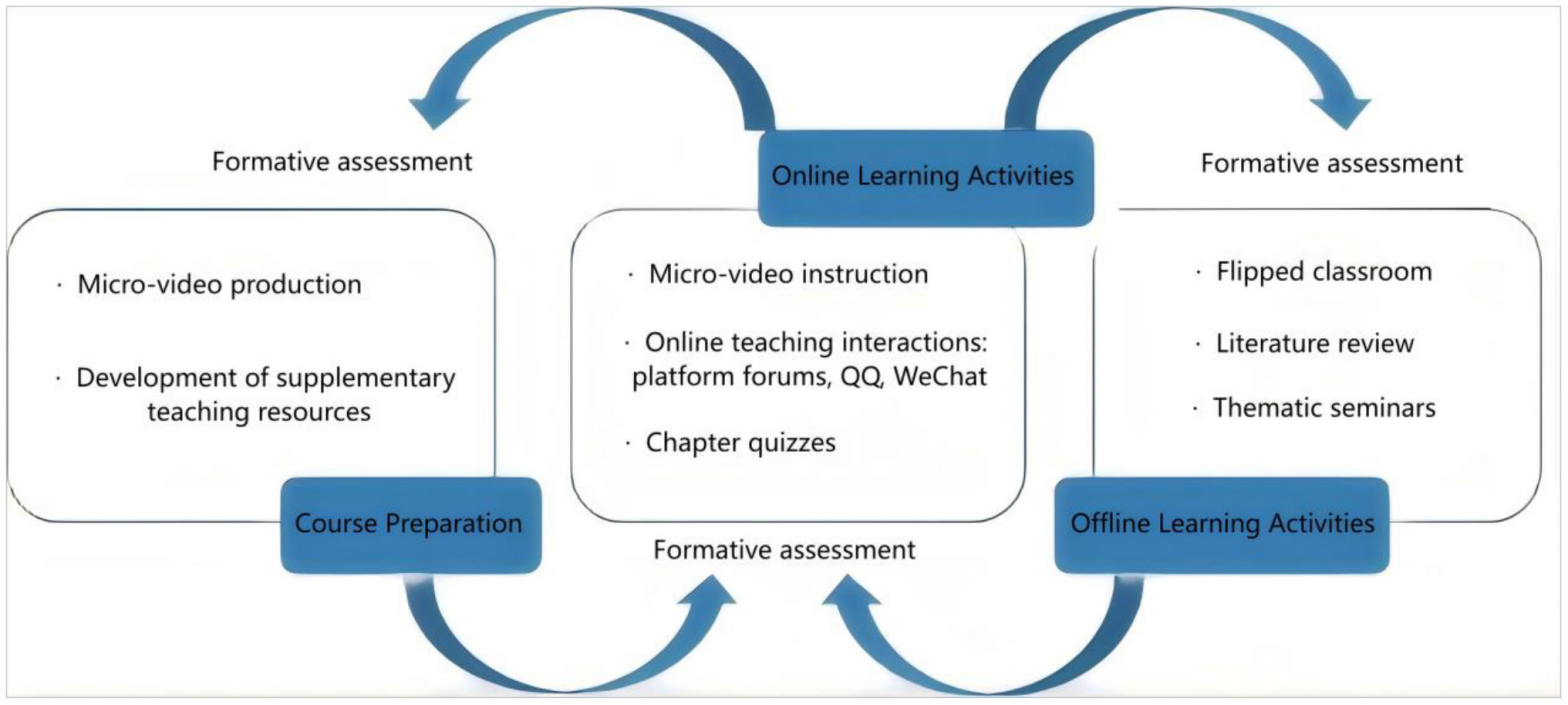
Schematic diagram of the SPOC-based blended teaching model.

The experimental group received an integrated, multicomponent educational package combining a SPOC-based blended teaching system with stage-specific, full-cycle training, as shown in Figure 2. The package included platform-based micro-video learning, online quizzes, face-to-face seminars, case-based collaborative learning, scenario simulation, practical skills training, instructor feedback, and continuous learning-process management. Teaching activities were implemented according to the educational objectives and curriculum guidelines. The intervention consisted of 3 sequential phases: preparation, core instruction, and consolidation. The specific teaching schedule is as follows: preparation (week 1): two 1-hour online training sessions for instructors and students to familiarize themselves with the platform. Core instruction (weeks 2–8): two blended-learning sessions per week, each lasting 90 minutes (45 minutes of online micro-video followed by a 45-minute face-to-face seminar). Consolidation (week 9): one 60-minute online quiz and one 60-minute in-person Q&A session.

The course frequency and duration are as follows: micro-video modules: 12 videos, each 8 to 12 minutes long. Online quizzes: six quizzes with 10 to 15 multiple-choice questions each, 20 minutes per quiz. Face-to-face seminars: groups of 8 to 12 students, 45 minutes per session. The SPOC teaching implementation: platform architecture: the SPOC teaching platform is based on a cloud computing architecture, integrating “Rain Classroom” and “Tencent Meeting.” “Rain Classroom” is used for pushing teaching materials, releasing quizzes, and statistics of learning data (e.g., video viewing progress, quiz completion rate), while “Tencent Meeting” is used for online live broadcasts and seminar interactions. The platform supports real-time data synchronization between web and mobile terminals and has functions such as learning progress tracking, interactive discussion areas, and automatic grading of objective questions. SPOC content development: all SPOC materials (micro-videos, case materials, quiz items) were custom-developed by the research team (composed of 3 senior imaging physicians with more than 10 years of clinical experience and 2 medical education experts). The content was developed in strict accordance with the “National Medical Imaging Internship Training Guidelines” and combined with the common clinical cases and technical characteristics of the Interventional Radiology Department. Instructor training: before the study, all 5 participating instructors received 8 hours of formal online pedagogy training, including SPOC platform operation (2 hours), online teaching activity design (2 hours), flipped classroom organization skills (2 hours), and online-offline integrated teaching experience sharing (2 hours). These implementation inputs were specific to the study setting and included access to 2 digital platforms, protected faculty training time, multidisciplinary expertise for content development, and ongoing instructor involvement in learning-data review, seminar facilitation, feedback, and course adjustment. The study did not prospectively quantify the time, workload, or financial cost associated with content development, platform maintenance, or intervention delivery.

The specific teaching content includes the following: pre-teaching phase: each intern will be required to download the “Rain Classroom” and “Tencent Meeting” apps on their mobile devices. The supervising instructors will push the teaching materials to these apps. Interns will use their free time to log into the apps for self-directed learning, which includes reviewing textbooks, searching for relevant literature, and completing online self-assessments. Instructors will extract learning data from the apps to summarize and analyze the effectiveness of the interns’ self-directed study. Based on the analysis, key and difficult areas of the training will be identified. Additionally, instructors will engage with interns through interactive discussions to address any questions or challenging points raised by the learners. In the teaching process, the supervising instructor first evaluates and summarizes the students’ independent learning outcomes based on learning data, providing guidance for their online learning. To facilitate the internalization of knowledge from mere storage, classroom learning is primarily centered around group-based collaborative learning through clinical case studies. The instructor pre-creates simulated clinical scenarios (e.g., a case of managing hemoptysis) and divides the students into groups of 4 to 5, appointing a group leader. The group leader guides the members in proposing individual perspectives on the case based on their existing knowledge, and through group discussions, they reach a consensus. The leader then assigns specific roles to each member, who engage in team-based learning through role-play or scenario simulation. The groups present their learning outcomes in various formats, such as research findings, case reports, role-plays, scenario simulations, or skill assessments. During inter-group discussions, each group selects one representative to present their viewpoints, while other group members may add supplementary information as needed. Finally, the supervising instructor summarizes the key points from the students’ discussions, provides feedback and corrections for any deficiencies identified during group discussions, and offers timely praise for particularly innovative ideas. After the teaching session, students log into the app to complete a survey assessing their mastery of the teaching content and submit a reflective learning report. The supervising instructor reviews the student data to reflect on the teaching content and methods, making timely adjustments as necessary. Once students begin clinical practice, they can access the app at any time to review the knowledge they have learned. The requirements for both online and offline learning are shown in Table 1.

**Table 1:** The design of teaching activities for both online and classroom modules.

Online module		Classroom module	
Teacher	Student	Teacher	Student
Publish course materials	Instructional video on key learning points	Organize classroom activities	Participate in topic debates
Online teaching management	Complete quizzes and assignments	Q&A on key and difficult points	Participate in themed presentations
Online Q&A	Discussion	Specialized lecture	Participate in other classroom activities

Q&A = question-and-answer.

### 2.5. Intervention adherence and fidelity monitoring

Intervention adherence was assessed using platform-generated learning records and instructor attendance logs. The Rain Classroom platform automatically recorded micro-video viewing progress and online quiz completion, while supervising instructors recorded attendance at face-to-face seminars. Video completion, quiz participation, and seminar attendance were summarized descriptively to evaluate whether the intervention was implemented and received as planned.

### 2.6. Evaluation metrics

Perceived job competency, self-directed learning ability, and teaching satisfaction were assessed using self-report questionnaires completed by the same participants at the immediate post-intervention time point. To reduce potential social desirability bias, the questionnaires were completed anonymously, and participants were informed that their responses would be used solely for research purposes and not affect their internship evaluation. Nevertheless, the shared respondent source, response format, and assessment time point could not eliminate response bias or common-method effects. The department-specific end-of-rotation examination was included as a performance-oriented educational outcome to complement the self-reported measures. However, because the examination was locally developed and administered within the study department, it was not regarded as an externally validated or fully objective measure of clinical competency.

#### 2.6.1. Job competency

The “Clinical Internship Physician Job Competency Rating Scale” was used to assess interns’ self-reported perceptions of their job competency. Originally developed by Wang et al, the questionnaire consists of 16 items across 4 dimensions: professional knowledge (4 items), professional skills (4 items), professional competence (4 items), and comprehensive qualities (4 items). Each item is rated using a 5-point Likert scale, with scores ranging from 1 to 5 points. The total score ranges from 16 to 80, with higher scores indicating higher perceived job competency rather than independently observed clinical performance. In Wang et al^[11]^ validation (N = 120), the overall Cronbach α was 0.89 (subscales: 0.82–0.88). In the present study, the total scale yielded α = 0.91, demonstrating excellent internal consistency.

#### 2.6.2. Self-directed learning ability

The “Self-Directed Learning Ability Scale” was used to assess participants’ self-reported perceptions of their self-directed learning ability. Developed by Liu and Zhang, the questionnaire consists of 61 items distributed across 4 dimensions: learning awareness (12 items), learning strategies (24 items), learning evaluation (12 items), and interpersonal skills (13 items). Each item is rated using a 5-point Likert scale, with scores ranging from 1 to 5 points. The total score ranges from 61 to 305, with higher scores indicating more favorable self-reported perceptions of self-directed learning ability. Liu and Zhang^[12]^ reported an overall Cronbach α of 0.94 (subscales 0.88–0.93). In the present study, we obtained α = 0.95 for the total scale and subscale alphas between 0.89 and 0.94.

#### 2.6.3. Teaching satisfaction

The “Teaching Satisfaction Rating Scale” was used to assess participants’ self-reported satisfaction with the teaching experience. Created by Chen et al, the questionnaire consists of 5 dimensions: satisfaction with the teaching plan, teaching methods, teaching environment, teaching content, and teaching duration. Each dimension is rated using a 5-point Likert scale, with scores ranging from 1 to 5 points. The total score ranges from 5 to 25, with higher scores indicating greater self-reported satisfaction with the teaching experience. Chen et al^[13]^ reported an overall α = 0.87; subscale alphas ranged from 0.80 to 0.85. In our dataset, the Cronbach α was 0.89 overall and 0.83 to 0.87 for subscales.

#### 2.6.4. End-of-rotation exam scores

After completion of the training, all interns underwent the same end-of-rotation examination under standardized conditions. The examination consisted of 2 components: a theoretical knowledge test and a practical skills assessment. The theoretical test was developed according to the departmental internship training objectives and a predefined content blueprint covering basic imaging knowledge, interventional radiology principles, image interpretation, and clinical case analysis. It included 50 multiple-choice questions and 5 case analysis questions, with a total score of 100 points. The same test paper, examination duration, and scoring criteria were applied to both groups.

The practical skills assessment focused on interventional procedural skills and image interpretation ability. A standardized scoring rubric was used to evaluate key domains, including procedural preparation, aseptic technique, basic interventional operation, image interpretation accuracy, clinical reasoning, and communication during simulated clinical scenarios. The assessment was independently scored by 2 senior physicians who were not involved in group allocation and were blinded to the interns’ group assignments. The final practical score was calculated as the mean of the 2 assessors’ scores. Inter-rater reliability for the practical skills assessment was 0.92, indicating good scoring consistency. The end-of-rotation examination was therefore regarded as a department-specific, performance-oriented educational outcome. Although identical examination content, administration procedures, scoring criteria, and blinded assessors were used for both groups, the examination was locally developed, had not undergone external validation, and was not considered a fully objective or broadly generalizable measure of clinical competency.

### 2.7. Statistical analysis

Statistical analyses were performed using SPSS version 29.0. Categorical variables are presented as n (%), and continuous variables are presented as mean ± standard deviation or median with interquartile range, as appropriate. Between-group comparisons of continuous outcomes were performed using Welch independent-samples *t* tests when heterogeneity of variance was present. Mann–Whitney *U* tests were additionally conducted as nonparametric sensitivity analyses.

To assess the robustness of the estimates in this small sample, nonparametric bootstrap confidence intervals were calculated using 20,000 resamples. An exploratory Holm correction was also applied to the 4 principal outcome comparisons to account for multiple testing.

Covariate-adjusted analyses were not performed because the participant-level baseline academic performance and digital literacy values required for reliable regression modeling were not available in the final analyzable dataset. These adjusted analyses had not been prespecified. Exploratory subgroup analyses were also not conducted because no subgroup hypotheses had been defined in advance and the sample size was insufficient for reliable stratified or interaction analyses. All tests were 2-sided, and *P* < .05 was considered statistically significant.

## 3. Results

### 3.1. Sensitivity analyses

Sensitivity analyses were performed to evaluate the robustness of the unadjusted between-group comparisons. The experimental group had higher scores than the control group for job competency, self-directed learning ability, teaching satisfaction, and end-of-rotation examination performance in both Welch independent-samples *t* tests and Mann–Whitney *U* tests. Nonparametric bootstrap analyses based on 20,000 resamples yielded confidence intervals that were consistent with the conventional estimates. Furthermore, all 4 principal between-group comparisons remained statistically significant after exploratory Holm correction for multiple testing. These analyses supported the direction and statistical robustness of the unadjusted findings, although they did not eliminate the possibility of residual confounding.

### 3.2. Self-reported perceived job competency scores of the 2 groups of radiology interns

At the immediate post-intervention assessment, the mean self-reportedjob competency score was 70.90 ± 2.01 in the experimental group and 49.63 ± 3.30 in the control group. The unadjusted mean difference was 21.27 points (95% confidence interval [CI], 19.85–22.68; *P* < .001). Because job competency was assessed using a participant-completed self-report scale, this difference represents perceived job competency rather than independently observed or objectively verified clinical performance, as shown in Table 2.

**Table 2:** Job competency scores of the 2 groups of radiology interns.

Group	Experimental group	Control group	*Z*	*P*	*r*
Professional knowledge	18 (17, 18)	12.5 (11, 13)	−6.789	<.001	0.876
Professional skills	18 (17, 18)	13 (11, 13)	−6.718	<.001	0.867
Professional competence	18 (17, 18)	12.5 (11, 13)	−6.755	<.001	0.872
Comprehensive qualities	18 (17.75, 18)	13 (12, 14)	−6.778	<.001	0.875
Total score	71 (69.75, 72.25)	49.5 (47, 51)	−6.675	<.001	0.862

### 3.3. Self-reported self-directed learning ability scores of the 2 groups of radiology interns

The mean self-reported self-directed learning ability score was 286.37 ± 4.90 in the experimental group and 194.50 ± 34.65 in the control group, corresponding to an unadjusted mean difference of 91.87 points (95% CI, 78.82–104.91; *P* < .001). These scores reflect participants’ perceptions of their learning awareness, strategies, evaluation, and interpersonal learning skills rather than directly observed learning behavior. The experimental-group mean represented approximately 93.9% of the maximum possible scale score, indicating that the potential influence of an upper-range or ceiling effect should be considered, as shown in Table 3.

**Table 3:** Self-directed learning ability scores of the 2 groups of radiology interns.

Group	Experimental group	Control group	*Z*	*P*	*r*
Learning awareness	56.5 (55.75, 57)	46.5 (35.75, 50)	−6.676	<.001	0.862
Learning strategies	113 (111, 114)	68.5 (61, 74.25)	−6.666	<.001	0.861
Learning evaluation	56 (55, 57.25)	42 (28.75, 48.5)	−6.567	<.001	0.848
Interpersonal skills	62 (60, 63.25)	49 (33.25, 54)	−6.652	<.001	0.859
Total score	285.5 (283, 290)	207 (151, 223.5)	−6.657	<.001	0.859

### 3.4. Self-reported teaching satisfaction scores and department-specific end-of-rotation examination scores of the 2 groups of radiology interns

The mean self-reported teaching satisfaction score was 24.00 ± 0.91 in the experimental group and 15.70 ± 1.21 in the control group, with an unadjusted mean difference of 8.30 points (95% CI, 7.75–8.85; *P* < .001). The experimental-group mean represented 96.0% of the maximum possible score, and 10 of 30 participants reached the maximum score of 25, indicating a marked ceiling pattern. The mean score on the department-specific end-of-rotation examination was 90.87 ± 2.60 in the experimental group and 86.23 ± 3.73 in the control group, corresponding to an unadjusted mean difference of 4.63 points (95% CI, 2.97–6.30; *P* < .001), as shown in Table 4. Teaching satisfaction represents participants’ perceptions of the educational experience, whereas the examination provides a locally developed, performance-oriented measure of short-term learning.

**Table 4:** Teaching satisfaction scores of the 2 groups of radiology interns.

Group	Experimental group	Control group	*Z/t*	*P*	*Cohen d/r*
Teaching plan	5 (5, 5)	3 (3, 4)	−6.893	<.001	0.890
Teaching methods	5 (4, 5)	3 (3, 4)	−6.387	<.001	0.825
Teaching environment	5 (4, 5)	3 (3, 3)	−6.630	<.001	0.856
Teaching content	5 (5, 5)	3 (3, 3)	−6.895	<.001	0.890
Teaching duration	5 (5, 5)	3 (3, 3)	−7.019	<.001	0.906
Total score	24 (23, 25)	16 (15, 16)	−6.742	<.001	0.870
End-of-rotation exam scores	90.870 ± 2.596	86.230 ± 3.730	5.585	<.001	1.44

### 3.5. Intervention adherence in the experimental group

In the experimental group, adherence to the intervention was generally high. The mean micro-video completion rate was 96.7%, and all interns completed at least 90% of the assigned micro-video modules. The online quiz participation rate was 100%, with all 30 interns completing the 6 scheduled quizzes within the required time window. The mean attendance rate for face-to-face seminars was 93.3%, and 28 of 30 interns attended all scheduled seminars. According to instructor teaching logs, all planned teaching sessions, case discussions, and seminar activities were implemented as scheduled. These findings support the feasibility and consistency of intervention implementation.

## 4. Discussion

Medical imaging is an interdisciplinary field that integrates theoretical knowledge, clinical reasoning, technical skills, and practical application. For medical students, internship training is essential for consolidating professional knowledge, developing imaging diagnostic thinking, and preparing for future clinical work.^[14]^ However, traditional internship teaching remains largely teacher-centered, with students often acting as passive recipients of knowledge. This approach may limit learning engagement, self-directed learning, and the practical application of knowledge.^[15,16]^ In this context, SPOC-based blended teaching provides a potential alternative by integrating online learning resources with offline seminars, flipped classroom activities, case-based discussions, and practical training.^[17,18]^ In the present study, 2 examples of SPOC-based teaching materials are provided as Supplemental Digital Content, Supplemental Digital Content 1. The illustrative case-based video module titled “Interventional Treatment of Hepatic Hemangioma” is provided in Supplemental Digital Content 1, Supplemental Digital Content 2, and the corresponding sample quiz items, including answer keys and explanations, are provided in Supplemental Digital Content 2, Supplemental Digital Content 3.

At the immediate post-intervention assessment, interns in the experimental group had higher scores than those in the control group across all evaluated outcomes. The between-group differences were particularly large for the self-reported measures of perceived job competency, self-directed learning ability, and teaching satisfaction, whereas the absolute difference in end-of-rotation examination performance was more modest. These findings indicate favorable between-group score differences at the single immediate post-intervention assessment. However, they provide no evidence regarding whether the differences persisted after the intervention or translated into long-term knowledge retention, independent workplace performance, clinical decision-making, or patient-related outcomes. The observed between-group differences should be attributed to the intervention package as a whole rather than to the SPOC platform, full-cycle training, or any other individual component. The magnitude of the observed differences requires cautious interpretation. Perceived job competency, self-directed learning ability, and teaching satisfaction were assessed using self-report questionnaires completed immediately after the intervention. These outcomes may therefore have been influenced by social desirability, common-method variance, participants’ awareness of receiving a novel teaching intervention, or short-term enthusiasm. In addition, the experimental-group scores for self-directed learning ability and teaching satisfaction approached the upper limits of their respective scales, and one-third of participants in the experimental group achieved the maximum teaching satisfaction score. This ceiling pattern may have compressed variability at the upper end of the scale and limited discrimination among participants with favorable responses. Consequently, the large numerical differences on these scales may overstate the magnitude of the intervention’s effect on enduring educational performance.

The statistical significance of the between-group differences should also be distinguished from their practical educational significance. The end-of-rotation examination provided a more performance-based outcome and showed an absolute mean difference of 4.63 points on a 100-point scale. Although this difference was statistically significant, the present study did not establish whether it corresponded to a meaningful change in subsequent workplace performance, independent clinical decision-making, patient care, or long-term knowledge retention. In contrast, the larger differences observed for perceived competency, self-directed learning, and satisfaction were derived from immediate self-assessment and should be interpreted primarily as differences in participants’ perceptions and learning experience. Accordingly, the findings support only the potential short-term educational value of the integrated package. They do not establish that the observed score differences were retained over time or resulted in durable or clinically meaningful improvements in professional performance.

It should also be noted that the intervention evaluated in this study was not a single-component SPOC intervention. Instead, it was an integrated educational package that included the SPOC platform, online micro-video learning, online quizzes, offline seminars, flipped classroom activities, case-based discussions, instructor feedback, and full-cycle stage-based training. Therefore, the observed improvements should be interpreted as theoverall effect of the bundled teaching system rather than the independent effect of the SPOC component alone. Although the SPOC platform provided the technological basis for flexible learning, process tracking, online assessment, and teacher–student interaction, its specific contribution cannot be separated from the accompanying offline activities and lifecycle-oriented instructional design in the present study.

Although the 2 groups were comparable in baseline academic performance and digital literacy, no additional covariate-adjusted analyses were performed. Thus, the present findings should be regarded as unadjusted short-term between-group differences rather than definitive evidence of an independent causal effect. Baseline academic performance and digital literacy may still influence students’ acceptance of online learning and subsequent learning outcomes, even when no statistically significant baseline imbalance is observed. Future studies with larger samples should prospectively collect relevant baseline variables and include adjusted analyses, such as analysis of covariance or multivariable regression, to further strengthen causal inference.

The nature of the outcome assessments further limits interpretation. Perceived job competency, self-directed learning ability, and teaching satisfaction were measured using similar Likert-type questionnaires completed by the same participants at the same immediate post-intervention time point. Although the instruments showed good internal consistency, internal consistency does not establish measurement objectivity or exclude systematic response bias. The shared respondent source, response format, and assessment context may have introduced common-method variance, while social desirability, expectancy, acquiescence, and novelty-related responses may have contributed to the consistently favorable pattern across the self-reported outcomes. These findings should therefore be interpreted primarily as short-term differences in participants’ perceived competency, learning ability, and teaching experience rather than direct evidence of improved objective clinical competency.

The end-of-rotation examination provided a more performance-oriented indicator of short-term learning. Both groups completed identical theoretical and practical assessments under consistent conditions, and the practical component was independently scored by 2 blinded senior physicians using a predefined rubric with good inter-rater reliability. Nevertheless, the examination was locally developed according to the objectives and content blueprint of a single department and had not undergone external validation or calibration against a broader competency standard. The 4.63-point between-group difference should therefore be interpreted as a difference in department-specific examination performance rather than definitive evidence of generalized clinical competency, independent workplace performance, or sustained learning. Future studies should incorporate externally validated examinations, objective structured clinical examinations, workplace-based assessments, independent external assessors, and longitudinal follow-up. Because no delayed post-intervention assessment was conducted, the trajectory and persistence of the observed differences remain unknown.

The preceding findings represent the outcomes directly observed in this study. The theoretical considerations discussed below are post hoc, hypothesis-generating interpretations and should not be regarded as empirically tested mechanisms.

Constructivist learning theory and self-determination theory may provide possible conceptual frameworks for considering how the integrated intervention could operate.^[19,20]^ Components such as micro-video learning, case-based discussion, scenario simulation, practical training, structured feedback, and peer–instructor interaction are conceptually compatible with active knowledge construction and with the self-determination constructs of autonomy, perceived competence, and relatedness. However, the present study did not directly measure cognitive engagement, knowledge-construction processes, learning motivation, behavioral engagement, autonomy, perceived competence as a motivational construct, or relatedness. It therefore cannot determine whether these variables changed during the intervention or mediated the observed between-group differences.

Accordingly, these theoretical explanations remain speculative and should be interpreted only as hypotheses for future research. Adequately powered studies should prospectively specify the proposed theoretical pathways, measure potential mediators using validated instruments at multiple time points, and use longitudinal mediation analyses to determine whether changes in motivation, engagement, or learning behavior precede and explain changes in educational outcomes.

A distinctive feature of the intervention package was the integration of SPOC-based blended learning with stage-specific full-cycle training. Unlike blended learning interventions that use relatively fixed online and offline arrangements throughout the training period,^[7]^ the present package organized teaching into preparation, core instruction, and consolidation phases. The preparation phase focused on platform familiarization and basic knowledge preview; the core instruction phase emphasized micro-video learning, case-based collaboration, seminars, and practical skills training; and the consolidation phase focused on knowledge integration, problem solving, assessment, and feedback. This stage-based organization may have helped align the overall teaching package with interns’ changing learning needs. However, because the components were delivered concurrently, the study cannot determine whether the observed outcomes were primarily attributable to the SPOC platform, stage-specific training, case-based learning, instructor feedback, or interactions among these elements.

The scalability and implementation feasibility of the intervention require careful consideration. Implementation in the present study was supported by 5 trained instructors, 8 hours of formal faculty training, customized learning materials developed by 3 senior imaging physicians and 2 medical education experts, access to established digital platforms, and continued faculty involvement in learning-data review, seminar facilitation, feedback, and instructional adjustment. These resources likely contributed to the high intervention adherence observed in this setting but may not be readily available in institutions with fewer faculty members, limited protected teaching time, restricted digital infrastructure, or less experience in blended learning.

The present study did not prospectively evaluate implementation cost, faculty workload, platform maintenance requirements, organizational readiness, instructor acceptability, or sustainability after the study period. Therefore, the high adherence achieved within this supported single-center environment should not be interpreted as evidence that the package can be implemented or scaled with similar fidelity in other institutions. Potential barriers to wider adoption include the initial workload required to develop high-quality micro-videos, cases, and quizzes; the need for faculty training and continuing technical support; scheduling of face-to-face activities alongside clinical duties; and differences in local curricula, learner characteristics, internet access, and institutional teaching policies.

Several strategies may improve scalability, although they were not tested in the present study. These include using existing low-cost institutional platforms, developing reusable and modular core teaching materials, adopting a train-the-trainer approach, introducing the package in phases, and allowing institutions to adapt case materials and schedules while retaining essential intervention components. Multicenter implementation studies should assess not only educational outcomes but also reach, adoption, fidelity, faculty workload, cost, acceptability, sustainability, and the extent of local adaptation required. Until such evidence is available, applicability beyond the present department should be considered uncertain.

Although instructors participated in platform training, resource development, case-based teaching, and feedback activities, the study did not evaluate whether these responsibilities improved faculty competence or instead increased workload and implementation burden. Any faculty development benefit, therefore, remains speculative. Future implementation studies should assess instructional competence, digital teaching literacy, teaching self-efficacy, workload, acceptability, and teaching satisfaction alongside student outcomes.

## 5. Conclusion

In conclusion, at the single immediate post-intervention assessment, medical imaging interns assigned to the integrated, multicomponent teaching package reported more favorable perceptions of job competency, self-directed learning ability, and teaching satisfaction and achieved higher scores on a department-specific end-of-rotation examination than those assigned to conventional teaching. These findings are restricted to short-term between-group score differences observed immediately after the intervention. Because no delayed assessments were conducted, the study cannot determine whether the observed differences were sustained, whether knowledge and skills were retained over time, or whether they translated into meaningful improvements in independent workplace performance, clinical decision-making, or patient care. The findings should, therefore, not be interpreted as evidence of durable or generalized clinical competency gains. In addition, the feasibility, faculty workload, cost, sustainability, and scalability of the package outside similarly supported institutions remain uncertain. Larger multicenter studies using externally validated assessments and longitudinal follow-up are required before conclusions can be drawn regarding long-term educational effectiveness or broader implementation.

### 5.1. Limitations

Several limitations should be considered. First, no formal a priori sample size calculation was performed, and the small single-center sample limits the precision and generalizability of the findings. Implementation occurred in a well-supported setting with trained faculty, customized teaching materials, and established digital platforms; resource requirements, faculty workload, cost, acceptability, and long-term sustainability were not formally evaluated, leaving scalability uncertain. Second, the intervention was delivered as an integrated, multicomponent package, preventing the independent contribution of the SPOC platform or any other component from being determined. Third, 3 principal outcomes were self-reported at a single immediate post-intervention time point and may have been affected by social desirability, common-method variance, novelty effects, and ceiling patterns. The department-specific end-of-rotation examination was locally developed and not externally validated, limiting its interpretation as a measure of generalized clinical competency. Fourth, covariate-adjusted analyses were not performed because participant-level baseline covariate data were unavailable. Although randomization and multiple sensitivity analyses supported the robustness of the unadjusted findings, residual imbalance and unmeasured factors, including motivation, prior online learning experience, and faculty–student rapport, cannot be excluded. Finally, the proposed psychological and learning-process mechanisms were not directly assessed, and no delayed follow-up was conducted to evaluate knowledge retention, workplace performance, patient-related outcomes, or faculty development. Adequately powered multicenter studies using prespecified adjusted analyses, externally validated objective assessments, and longitudinal follow-up are warranted.

## Author contributions

**Conceptualization:** Junjie Chen, Jun Cui, Xin Li.

**Data curation:** Jiuxiao Yang, Xiao Yang, Hui Zhong, Fei Wang, Qingwen Gu, Cailiang Duan.

**Funding acquisition:** Junjie Chen.

**Writing – original draft:** Junjie Chen.







## References

[1] Zeng J Liu L Tong X. Application of blended teaching model based on SPOC and TBL in dermatology and venereology. BMC Med Educ. 2021;21:606. 34879860 10.1186/s12909-021-03042-7 PMC8656105

[2] Gao B Yan C An Y Guo X. Research on mixed SPOC and WebQuest modes in clinical teaching. Asian J Surg. 2024;47:4235–6. 38797585 10.1016/j.asjsur.2024.05.081

[3] Shen G An Y. Influencing factors of psychological stress under the mixed teaching mode based on SPOC+PBL. Front Psychol. 2022;13:979206. 36148096 10.3389/fpsyg.2022.979206 PMC9486209

[4] National Health Commission. Notice on the issuance of the “14th five-year plan for national clinical specialty capacity building” [EB/OL]. Bull Natl Health Commission People’s Republic China. 2021:20–7.

[5] Luo J Cheng R Lei L Cheng L Yang Y Hu T. Science popularization education regarding oral health-general health for nonmedical undergraduates applying a SPOC teaching model. Dis Markers. 2022;2022:3439509. 35783016 10.1155/2022/3439509 PMC9247851

[6] Kang HY Kim HR. Impact of blended learning on learning outcomes in the public healthcare education course: a review of flipped classroom with team-based learning. BMC Med Educ. 2021;21:78. 33509176 10.1186/s12909-021-02508-y PMC7845047

[7] Li S Su L Lou R Liu Y Zhang H Jiang L. Blended teaching mode based on small private online course and case-based learning in analgesia and sedation education in China: a comparison with an offline mode. BMC Med Educ. 2024;24:28. 38178081 10.1186/s12909-023-04839-4 PMC10768422

[8] Wang H Zhang W Kong W. The effects of “small private online course + flipped classroom” teaching on job competency of nuclear medicine training trainees. BMC Med Educ. 2024;24:1542. 39731074 10.1186/s12909-024-06579-5 PMC11681664

[9] Qiu H Li Q Li C. How technology facilitates tourism education in COVID-19:case study of nankai University. J Hosp Leis Sport Tour Educ. 2021;29:100288. 34720752 10.1016/j.jhlste.2020.100288 PMC8542758

[10] Sun S Li Q Yang J. Application of the “SPOC + Flipped Classroom” model in oncology imaging teaching. Chin Continuing Med Educ. 2022;14:73–7.

[11] Wang H Li Y Chen Z. Development and validation of the clinical internship physician job competency rating scale. J Med Educ. 2019;15:210–8.

[12] Liu X Zhang Y. Construction and psychometric evaluation of a self-directed learning ability scale for health-profession students. Adv Health Sci Educ. 2017;22:505–17.

[13] Chen J Wu L Huang M. Teaching satisfaction rating scale: design and validation in clinical education. Clin Teach. 2018;15:145–50.28474405

[14] Alshamrani KM Alkenawi AA Kaifi RE. The barriers, motives, perceptions, and attitudes toward research among radiology practitioners and interns in Saudi Arabia: a cross-sectional study. Front Med (Lausanne). 2023;10:1266285. 37877018 10.3389/fmed.2023.1266285 PMC10593452

[15] Zhao C Xu T Yao Y Song Q Xu B. Comparison of case-based learning using Watson for oncology and traditional method in teaching undergraduate medical students. Int J Med Inform. 2023;177:105117. 37301132 10.1016/j.ijmedinf.2023.105117

[16] Xu Y Wang L Li P. Exploring the impact of online and offline teaching methods on the cognitive abilities of medical students: a comparative study. BMC Med Educ. 2023;23:557. 37553632 10.1186/s12909-023-04549-x PMC10410817

[17] Lu YM Mo XP He X. Application of SPOC-based blended teaching model in medical imaging teaching for traditional Chinese medicine majors. Youjiang Med. 2025;53:275–9.

[18] Gong XJ Hou LY Liu YW. Application of the SPOC combined with the “Five-step” teaching method in literature discussion courses for master’s nursing students. Nurs Res. 2023;37:3382–7.

[19] Shi XY Lu BR Yin Q Wang Q-W Fang Y-Q Sun Z-G. Whether case-based teaching combined with the flipped classroom is more valuable than traditional lecture-based teaching methods in clinical medical education: a systematic review and meta-analysis. BMC Med Educ. 2025;25:906. 40598010 10.1186/s12909-025-07465-4 PMC12210433

[20] Riebner N Sonnauer F Rauch M. Promoting motivation and reducing stress in medical students by utilizing self-determination theory – a randomized controlled trial in practical psychiatry courses. BMC Med Educ. 2024;24:1177. 39427147 10.1186/s12909-024-06181-9 PMC11491017

