# Supplementary material for: Application of a SPOC-based blended teaching system in full-cycle training for medical imaging interns: A randomized controlled educational intervention study
Source: Medicine (Baltimore). 2026 Jul 24;105(30):e49908. doi: 10.1097/MD.0000000000049908 (PMC13406057; doi:10.1097/MD.0000000000049908)
Supplement: Supplementary file 2 [file medi-105-e49908-s002.docx]

**Supplemental Digital Content 1:** Illustrative case-based video module (Theme: Interventional Treatment of Hepatic Hemangioma).

1. Video outline: (1) Overview of hepatic hemangioma (epidemiology, clinical manifestations); (2) Imaging diagnosis of hepatic hemangioma (ultrasound, CT, MRI features); (3) Indications and contraindications of interventional treatment for hepatic hemangioma; (4) Surgical process of transcatheter arterial embolization (TAE) for hepatic hemangioma; (5) Postoperative complications and management. (2) Key knowledge points: CT/MRI typical manifestations of hepatic hemangioma, TAE surgical steps, postoperative complication prevention. (3) Clinical scenario design: A 45-year-old female patient was found to have a hepatic space-occupying lesion during physical examination. Ultrasound suggested hepatic hemangioma, and further enhanced CT was performed to confirm the diagnosis. The patient was admitted for interventional treatment. The video includes simulated surgical operation demonstrations and postoperative care process explanations.
