# Supplementary material for: Application of a SPOC-based blended teaching system in full-cycle training for medical imaging interns: A randomized controlled educational intervention study
Source: Medicine (Baltimore). 2026 Jul 24;105(30):e49908. doi: 10.1097/MD.0000000000049908 (PMC13406057; doi:10.1097/MD.0000000000049908)
Supplement: Supplementary file 3 [file medi-105-e49908-s003.docx]

**Supplemental Digital Content 2:** Sample quiz items for the above video module (with answer keys and explanations).

Which of the following is the typical CT enhancement feature of hepatic hemangioma? ( )

A. Peripheral nodular enhancement, progressive centripetal filling

B. Uniform enhancement in the arterial phase

C. No enhancement in the venous phase

D. Rim enhancement only

Answer: A. Explanation: The typical CT enhancement feature of hepatic hemangioma is peripheral nodular enhancement in the arterial phase, followed by progressive centripetal filling in the venous phase and delayed phase, which is known as the "progressive filling sign".
